# Mechanical Properties of Electrospun, Blended Fibrinogen: PCL Nanofibers

**DOI:** 10.3390/nano10091843

**Published:** 2020-09-15

**Authors:** Jacquelyn M. Sharpe, Hyunsu Lee, Adam R. Hall, Keith Bonin, Martin Guthold

**Affiliations:** 1 Department of Physics, Wake Forest University, Winston-Salem, NC 27109, USA; jmsharpe98@gmail.com (J.M.S.); leeh313@wfu.edu (H.L.); bonin@wfu.edu (K.B.); 2 School of Biomedical Engineering and Sciences, Wake Forest School of Medicine, Virginia Tech-Wake Forest University, Winston-Salem, NC 27101, USA; arhall@wakehealth.edu; 3 Comprehensive Cancer Center, Wake Forest School of Medicine, Winston-Salem, NC 27157, USA; 4 Center for Functional Materials, Wake Forest University, Winston-Salem, NC 27109, USA

**Keywords:** electrospinning, fibrinogen, poly-ε-caprolactone, mechanical characterization

## Abstract

Electrospun nanofibers manufactured from biocompatible materials are used in numerous bioengineering applications, such as tissue engineering, creating organoids or dressings, and drug delivery. In many of these applications, the morphological and mechanical properties of the single fiber affect their function. We used a combined atomic force microscope (AFM)/optical microscope technique to determine the mechanical properties of nanofibers that were electrospun from a 50:50 fibrinogen:PCL (poly-ε-caprolactone) blend. Both of these materials are widely available and biocompatible. Fibers were spun onto a striated substrate with 6 μm wide grooves, anchored with epoxy on the ridges and pulled with the AFM probe. The fibers showed significant strain softening, as the modulus decreased from an initial value of 1700 MPa (5–10% strain) to 110 MPa (>40% strain). Despite this extreme strain softening, these fibers were very extensible, with a breaking strain of 100%. The fibers exhibited high energy loss (up to 70%) and strains larger than 5% permanently deformed the fibers. These fibers displayed the stress–strain curves of a ductile material. We provide a comparison of the mechanical properties of these blended fibers with other electrospun and natural nanofibers. This work expands a growing library of mechanically characterized, electrospun materials for biomedical applications.

## 1. Introduction

Electrospinning is a relatively straightforward method to make fibers with micrometer and sub-micrometer diameters from many different materials. The small size and high material diversity have made electrospun fibers an attractive choice for numerous, novel applications in a variety of fields, including filtration, catalysis, energy harvesting and storage, and biomedicine [1]. In electrospinning, a viscous solution that may consist of a mixture of materials is slowly pumped out of a small orifice and subjected to an electric field. The electric field stretches the emerging solution into small fibers. The fibers are collected at a grounded collection site. The technique of electrospinning was first reported and patented in the early 20th century [2,3]; however, initially it was only used in a few applications relating to textiles [4] and filtering [1]. This initial sparsity of applications has been attributed to a lack of tools to characterize and, thus, understand these nanoscopic fibers [1]. The theoretical underpinning of electrospinning was developed in the late 1960 by Geoffrey Taylor [5], and characterization tools, such as powerful microscopes, became more commonplace in the 1990s. Once a basic theoretical understanding of electrospinning was developed and fibers could be characterized, electrospinning saw an enormous increase in applications, especially over the last two decades. Significant research efforts in developing innovative applications, thorough fiber characterization, and improved theoretical understanding of fiber formation and properties are ongoing [1].

One specific area, in which electrospun fibers are finding widespread use, is biomedicine. This is due to several attributes that make electrospun fibers quite suitable for applications in this field. First, fibers can be electrospun from many materials that are biocompatible, such as nontoxic plastics, proteins and chemicals. Second, molecules that provide a desired action can be mixed into the spinning solution or can be attached to the fibers after spinning. These molecules include cellular growth factors to control cell growth [6,7,8,9], drugs to suppress coagulation [10], therapeutics to treat cancer [11], or molecular cocktails to control wound healing and angiogenesis [12]. Third, networks made from electrospun fibers can be fabricated so that they mimic the fiber dimensions, architecture and composition of the extracellular matrix. Recent research, that utilized scaffolds made from electrospun fibers, showed that cell growth and migration are affected by the fiber diameter and fiber alignment of the scaffold [9,12,13,14,15,16]. Fourth, the mechanical properties of the electrospun fibers and mats can be controlled by using different materials and/or by chemically crosslinking the fibers [17,18,19,20].

Due to these favorable properties, electrospun fibers have been used in numerous biomedical applications that include scaffolds for tissue engineering (for reviews, see [1,21], in particular vascular tissue (blood vessels) [7,8,10,14,22,23,24], skin [25] and cardiac tissue [26,27]; wound healing and wound dressing [12]; and drug delivery constructs [11]. Additional emerging applications involve tactile sensing and flow sensing utilizing electrospun, piezoelectric polyvinylidene fluoride nanofibers [28,29].

The mechanical properties of electrospun fibers play a critical role in the performance of the devices made from these fibers. Obviously, at a macroscopic level, a device needs to have a certain strength and stability so that it can be handled. The macroscopic strength of a device derives from the strength of the individual fibers that make up the device, the connection between the fibers and the device architectures (e.g. density of fibers, alignment of fibers). More subtly, the mechanical properties of the individual fibers can also be important at the microscopic level. For example, it was shown that the stiffness of the cell substrate can influence the growth and differentiation of stem cells [30], and, indeed, it was also found that the mechanical properties of electrospun fibers can influence cell growth and migration [17,18,19,20]. Thus, the mechanical properties of electrospun fibers affect device performance at the macroscopic and microscopic level.

Devices for different applications demand different mechanical properties, and some applications may even require a change in mechanical properties as the device changes environments. Most devices are used, stored or manufactured in a dry environment, such as patches, dressings, sutures, pills or scaffolds. Thus, the mechanical properties of devices in a dry environment are relevant. Many of these devices may then be exposed to fluids. For many devices, it is important that they are stable in a dry environment, but soften or dissolve in a wet environment. For example, this may be desirable for drug delivery devices, sutures and scaffolds. Thus, for most devices the mechanical properties in a dry and wet state are relevant to their function. The single fibers that comprise the device critically define the overall mechanical properties and integrity of the device. 

Mechanical measurements on individual electrospun fibers are technically challenging because of their small size. These measurements require techniques that can determine nanometer displacements and nanonewton forces. The atomic force microscope (AFM), meets these requirements. Our lab has developed a technique to perform mechanical measurements on nanofibers using a combined AFM/optical microscope technique. In these AFM-based measurements, the fibers are suspended over micrometer-sized grooves and then mechanically manipulated with the AFM probe. During this procedure, the force on the fiber and its displacement are measured, while the fiber is observed with an optical microscope that is situated underneath the sample [31,32,33,34].

Previously, we determined the properties of electrospun nanofibers formed from pure fibrinogen, collagen and poly-ε-caprolactone (PCL) [31,32,33,34]. A next step is expanding the material library by blending materials to create novel fibers with a range of properties. Our overall goal is to create a library of mechanically characterized, electrospun nanofibers that are suitable for biomedical applications (i.e., that are made from biocompatible materials). Different applications may require fibers with different mechanical properties. Expanding the library of materials gives researchers a choice to select a mechanically characterized fiber that is suitable for their application. 

PCL is a synthetic polymer. It is widely available, inexpensive, biocompatible and approved for use in humans. Electrospun fibers made from pure PCL (molecular weight, 120,000–300,000 g/mol)) have a modulus of 62 MPa and a large extensibility of 98% stain, or about twice their initial length [31]. They can be stretched elastically (without permanent deformation to about 20% strain). PCL has a slow degradation time—about one year—when used in the human body.

Fibrinogen is a naturally occurring blood protein (MW, 340,000) that, upon activation, polymerizes into a network of 100–200 nm-thick fibrin fibers. This network is the major structural and mechanical component of a blood clot. Dry, electrospun fibrinogen fibers have a modulus of 4200 MPa—much higher than PCL fibers, and an extensibility of 110%. They can be stretched elastically to 16% strain [32]. Wet, electrospun fibrinogen fibers are significantly softer and have a modulus of 18 MPa, and an extensibility of 130% [34].

With the knowledge of the mechanical properties of the pure, electrospun PCL and fibrinogen fibers, we created a new fiber type by combining these two materials into a 50:50 blend. This combination of natural and synthetic materials expands the library of fibers available to scientists. We studied these fibers using an atomic force microscope (AFM) technique, and determined the total and elastic modulus, maximum stress and strain, and energy loss. The blended 50:50 fibers show mechanical properties that are a blend of the properties of the pure fibrinogen and PCL fibers. They have a modulus between that of the fibrinogen and PCL fibers, and a similar breaking strain of about 100%. They also show extreme strain softening and very high energy loss in cyclical stress–strain curves. This indicates that the blended material mostly deforms plastically and that the fiber components slide past each other with little elastic connections between them.

## 2. Materials and Methods

### 2.1. Preparation of 50:50 Fibrinogen:PCL Solution

First, stock solutions of fibrinogen and poly-epsilon-caprolactone (PCL) were prepared. PCL pellets (average molecular weight MW 80,000 g/mol, 440744-5G, Sigma-Aldrich, St. Louis, MO, USA) were mixed with Hexafluoro-2-propanol (HFP, Sigma-Aldrich, St. Lois, MO, USA) at a concentration of 100 mg/mL. This mixture was stirred with a magnetic stir-bar for several hours until a viscous (honey-like), homogeneous liquid was obtained (visual inspection), and the PCL pellets were fully dissolved. To create the fibrinogen solution, bovine fibrinogen powder (>85% clottable, final concentration 100 mg/mL, Sigma-Aldrich, St. Lois, MO, USA) was mixed with a solvent consisting of 9 parts HFP and 1-part Dulbecco’s Modified Eagle’s Medium (11960, Thermo Fisher Scientific, Waltham, MA, USA). The solution was stirred for several hours using a magnetic stir bar, creating a milky, homogenous solution (visual inspection) slightly more viscous than water. The solutions can be stored at 4 °C for at least several days without noticeable changes in homogeneity (no separation or clumping). To create the 50:50 fibrinogen:PCL fibers, 1 mL of the PCL solution and 1 mL of the fibrinogen solution were mixed in a beaker with a stir bar until well blended. The solution may be stored for several days at 4 °C. 

### 2.2. Preparation of Striated Substrate

The preparation of the striated substrate is based on the soft lithography and micromoulding techniques as generally described in [35], and specifically applied to electrospun nanofiber mechanical measurements in [31,32,33,34]. First, a PDMS (polydimethylsiloxane) stamp (negative (inverted) relief structure of the striated substrate) was created by pouring dimethyl siloxane mixed with a catalyst (Sylgard^®^ 184, Sigma-Aldrich, St. Louis, MO, USA) onto an SU-8-silicon master grid in a large plastic Petri dish. A 1 cm × 1 cm stamp can then be excised with a scalpel. Excised stamps were stored in a 2% sodium dodecyl sulfate (SDS) solution to keep them clean; the stamps can be stored for at least several months and used repeatedly. To create the striated substrate, a drop of Norland Optical Adhesive-81 (NOA-81, Norland Products, Cranbury, NJ, USA) was placed on a 60 mm × 24 mm, #1.5 microscope cover slide (Thermo Fisher Scientific, Waltham, MA, USA). The PDMS stamp was pressed into the NOA-81 drop on the slide and cured with 365 nm UV light (Benchtop 3UV transilluminator, UVP, Upland, CA, USA) for several minutes. The substrate had ridges of width 7.3 μm and height 6 μm. The gaps between the ridges were 6 μm across. 

### 2.3. Electrospinning of 50:50 Fibrinogen:PCL Fibers

**Figure 1:**
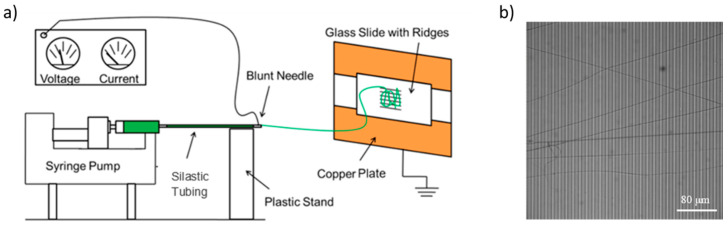
(**a**) Schematic of the electrospinning setup. The electric potential difference between the needle and the collection site is set to 20,000 V. Figure adapted from [3]. (**b**) Optical microscopy image of electrospun fibers on the striated substrate. Ridges (width, 7.3 μm; height, 6 μm) appear in darker gray; gaps (6 μm across) appear in lighter gray.

About 2 mL of the previously prepared 50:50 fibrinogen:PCL solution were pipetted into a 5 mL syringe that was outfitted with a 20-gauge, 1 inch long, blunt syringe needle (#901-20-100, CML supply, Lexington, KY, USA), and placed in a syringe pump (PHD 2000 Infusion syringe pump, Harvard Apparatus, Holliston, MA, USA). The needle of the syringe was connected to about 10 cm of Dow Corning silastic medical-grade tubing with an inner diameter of 0.03” and an outer diameter of 0.065” (Dow Corning, Midland, MI, USA). This tubing was connected to another blunt needle (same type) and a stream of solution was generated at a rate of 0.8 mL/hr. The blunt needle at the end was connected to an electric potential of 20,000 V (CZE1000R, Spellman High Voltage Electronics Corporation, Hauppauge, NY, USA) via an alligator clip. The blunt needle is separated by 15 cm from the collection site, where the striated substrate is mounted. The collection site is connected to ground (0 V). The collection site consists of two grounded copper plates separated by 2 cm. The glass cover slip with striated substrate was taped with conducting copper tape on top of the copper plates. Two strips of copper tape were used so that the fibers are whipped back and forth across the glass slide and align across the ridges [36,37,38]. A schematic diagram of the setup is shown in Figure 1. Fibers were prepared in a home-built Plexiglas glove box in an air-conditioned room (24 °C, 55% relative humidity). Temperature and humidity likely affect fiber formation and we, therefore, controlled them. 

### 2.4. Anchoring of Fibers to Ridges

In initial fiber pulling experiments, we found that the fibers were not sufficiently attached to the ridges as they moved across the ridge when pulled by the AFM probe. Thus, once the fibers were spun, they had to be anchored to the ridges. A drop of about 10 μL of white Marine Loctite Epoxy (Henkel Corporation, Rocky Hill, CT, USA) was placed on the slide next to the substrate. This epoxy was chosen because, according to the manufacturer, it does not shrink and is resistant to water and most common solvents. Using a used, dulled AFM tip, a small amount of epoxy was picked up by the tip by dipping the tip into the epoxy. Using the Asylum AFM controls, the tip was lowered onto the ridge next to a fiber, such that glue was transferred from the tip to the ridge, gluing the fiber to the ridge. Once a selection of about 15 fibers was glued, the sample was left to cure overnight. This method can be likened to a dip-pen, in which the pen is dipped into an inkwell and the liquid is transferred to the writing surface. On the nanoscale, this process has been termed dip-pen nanolithography (DPN) [39]. 

### 2.5. Combined AFM/Optical Microscopy

To test the fibers’ mechanical properties, the sample was placed on the stage of the combined optical microscope/atomic force microscope (AFM) stage (inverted optical microscope, Olympus IX73, Olympus America, Inc. Center Valley, PA, USA; AFM, MFP-3D-Bio, Oxford Instruments-Asylum Research, Santa Barbara, CA, USA). The stage was adjusted so that the sample is situated above the objective lens and underneath the AFM probe. The selected fibers were then scanned on the ridges using the AC mode (tapping mode) of the AFM. These scans provided the fiber diameter; image analysis was performed using Gwyddion software (http://gwyddion.net/) (Brno, Czech Republic). Subsequently, the AFM was switched to contact mode for fiber manipulations. Initially, the probe was raised well above a particular fiber. The AFM probe was then lowered in 500 nm steps followed by a small test movement in the x-y-position to see if the AFM probe was at the horizontal level of the fiber. Once the horizontal level of the fiber was reached, the lateral x-y-position was adjusted until the probe aligned with the center of a particular fiber. This overall alignment procedure ensured that the AFM probe was aligned with the center of a fiber, and that the fiber contacted the AFM probe at the very end of the tip. The fiber was then horizontally pulled, in a direction perpendicular to the fiber length, at a speed of 300 nm/s until it broke. Care was taken to ensure that the sample and the AFM were level. Asylum Research AFM Probes AC160TSA were used (nominal properties: length, L = 160 μm, width, w = 40 μm, height h = 14 μm, resonance frequency f_0_ = 300 kHz and spring constant, k = 26 N/m). The cantilever thickness, t, which was determined from calculations as outlined in detail below, was on average 4 μm.

We also conducted incremental stress–strain curves on the fibers. For these incremental stress–strain curves, the fiber was pulled with a rate between 30 and 50 nm/s for 20 s, and then allowed to relax for 30 s (tip stopped pulling for 30 s). This process was repeated until the fiber broke, typically four or five sequences.

Additionally, the energy loss of the fibers was tested by pulling the fibers to a small strain and returning the tip to the starting position. These cyclical pulls (forward and back) were repeated several times on a single fiber, with strains usually starting at 10% and increasing by 10% each time until the fiber ruptured.

The AFM recorded the time in seconds, the position in micrometers, and the lateral force in Volts (photodiode signal) for the duration of the tests. The raw data were converted to stress in MPa, and strain in percentage, by the procedure given below.

Fiber mechanical measurements were performed in an air-conditioned room (24 °C, 55% relative humidity. Temperature and humidity likely affect mechanical properties and were therefore controlled.

### 2.6. AFM Force Measurements

**Figure 2:**
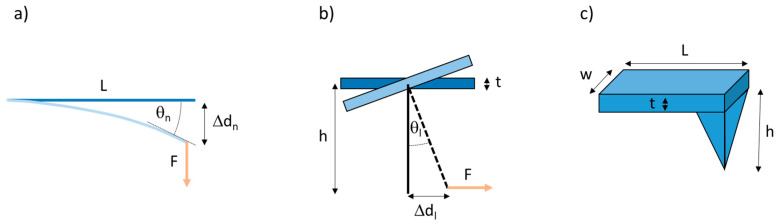
Geometry for normal and lateral atomic force microscopy (AFM) force measurements. (**a**) Side view of cantilever. A normal force applied to an AFM cantilever results in a normal deflection of the cantilever (treated like a flexible beam) by a distance Δd_n_, and a bend angle θ_n_ (measured tangentially at the end of the cantilever). (**b**) Frontal view of cantilever. A lateral force applied to an AFM cantilever results in a lateral deflection of the cantilever by a distance Δd_l_, and a bend angle θ_l_. This is the situation when pulling on a fiber. (**c**) Schematic diagram of an AFM probe showing the length (L), width (w) and thickness (t) of the cantilever and the height (h) of the AFM tip.

The AFM uses a microscopic tip attached perpendicularly to a cantilever to image or mechanically manipulate samples. The AFM can apply a normal force, pressing straight down on the sample or pulling straight up on the (surface-anchored) sample. It can also apply a lateral force, as the tip can laterally pull on microscopic materials, such as fibers. When a force, F, is applied to the cantilever, it deflects the cantilever by a distance Δd, and, according to Hooke’s law, the magnitude of the force is F = k·Δd, where k is the spring constant of the cantilever. Based on beam mechanics [40], for a normal force measurement, (Figure 2a), the normal spring constant is given by

(1) $$k_{n}=\frac{Ewt^{3}}{4L^{3}}$$

Likewise, for a lateral force measurement, (Figure 2b), the lateral spring constant is given by

(2) $$k_{l}=\frac{Gwt^{3}}{3L{\left(h+\frac{t}{2}\right)}^{2}}$$

where *L*, *w* and *t* are the length, width and thickness of the cantilever, *h* is the tip height (Figure 2), and E and G are the Young’s and shear modulus of the cantilever (E = 1.69·10^11^ Pa; G = 0.5·10^11^ Pa). 

The AFM records the deflection of the tip by reflecting a laser off the cantilever and recording the change in light distribution in a four-quadrant photodiode. A normal force measurement results in a change in the normal photodiode signal, ΔV_n_ = V_top_ − V_bottom_, and a lateral force measurement results in a change in the lateral photodiode voltage signal, ΔV_l_ = V_left_ − V_right_.

A normal or lateral deflection of the cantilever results in a change in voltage output of the photodiode, Δ*V_n_* or Δ*V_l_.* To convert voltage data and the data of the tip’s position into force–distance or stress–strain curves, the following procedure is performed. 

The normal voltage signal, Δ*V_n_*, is proportional to the deflection, Δ*d_n_*, of the cantilever; thus, Hooke’s law in the normal direction, *F_n_ = k_n_·*Δ*d_n_*, becomes

(3) $$F_{n}=\;k_{n}S_{n}\Delta V_{n}$$

where $S_{n}=\frac{\Delta d_{n}}{\Delta V_{n}}$ is the normal sensitivity, the conversion factor of the normal tip displacement, Δ*d_n_*, to the normal voltage signal, Δ*V_n_.* This quantity is determined before each experiment by taking a force curve on a hard surface, deflecting the tip by a known amount Δ*d_n_* and recording the corresponding voltage signal Δ*V_n_*. Alternatively, it may be determined via an Asylum software routine from the cantilever resonance frequency. 

Using Equation (1), Equation (3) becomes

(4) $$F_{n}=\;\frac{Ewt^{3}}{4L^{3}}S_{n}\Delta V_{n}$$

which is used to determine the force in the normal force measurements from the voltage signal Δ*V_n_*.

Hooke’s law in the lateral direction, *F_l_ = k_l_*Δ*d_l_*, becomes

(5) $$F_{l}=\;k_{l}\cdot S_{l}\cdot \Delta V_{l}$$

where $\;S_{l}=\frac{\Delta d_{l}}{\Delta V_{l}}$ is the lateral sensitivity, the conversion factor of the lateral tip displacement, Δ*d_l_*, to the lateral voltage signal Δ*V_l_*. This quantity is derived from the normal sensitivity as follows. 

According to beam mechanics, the bend angle, $\theta_{n}$, of a flexible cantilever of length *L*, that is fixed at one end and deflected at the other end a distance, Δ*d_n_*, is given by $\theta_{n}=\frac{3}{2}\times \frac{\Delta d_{n}}{L}\;$. The photodiode output signal of the AFM in the normal direction, Δ*V_n_*, is proportional to the bend of the cantilever

(6) $$\Delta V_{n}\propto \Delta \theta_{n}=\;\frac{3}{2}\cdot \frac{\Delta d_{n}}{L}$$

**Figure 3:**
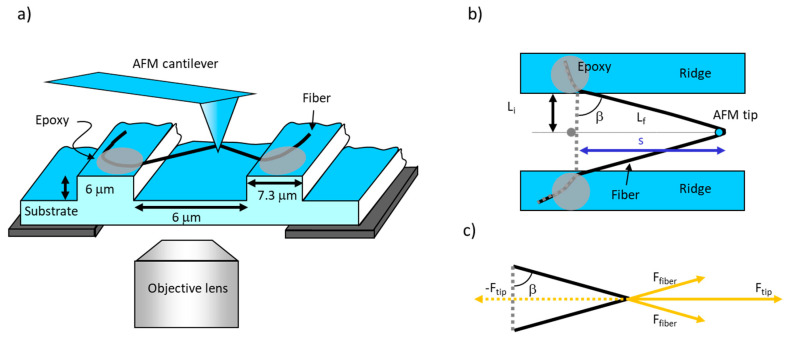
Schematic of fiber manipulation. (**a**) Side view. The fiber is suspended over the grooves and pulled by the laterally moving AFM probe. The fiber is anchored to the ridges by epoxy (gray ellipses). The fiber can be viewed by the inverted microscope beneath the substrate. (**b**) Top view. *L_i_* is half of the initial full length of the fiber while *L_f_* is half of the stretched length, assuming the fiber is pulled in the middle. The angle between *L_i_* and *L_f_* is *β*, and *s* is the distance travelled by the AFM tip. Figure adapted from [31]. (**c**) Force diagram showing the force balance between the force applied by the tip on the fiber, *F_tip_*, and the force applied by the fiber on the tip, −*F_tip_*. These forces are equal and opposite according to Newton’s third law. *F_tip_* is the sum of the x-components of the two *F_fiber_* forces that are applied to the top and the bottom arm of the fiber. *F_fiber_* is applied along the length of the fiber. *F_tip_* is equal in magnitude to the lateral force measured by AFM, *F_l_*; *F_fiber_* is the force that stretches the upper and lower arm of the fiber (assuming symmetric pulling in the middle of the fiber).

If the cantilever is deflected laterally by a distance, Δ*d_l_*, (which can be approximated by the arc length, Figure 3), the lateral deflection angle, $\theta_{l}$, is $\theta_{l}=\;\frac{\Delta d_{l}}{\left(h+\;\frac{t}{2}\right)}$. Here, it is assumed that the cantilever tip is stiff (i.e., does not bend). The photodiode output signal of the AFM in the lateral direction, $\Delta V_{l}$, is proportional to the bend angle of the cantilever,

(7) $$\Delta V_{l}\propto \Delta \theta_{l}=\;\frac{\Delta d_{l}}{\left(h+\frac{t}{2}\right)}$$

Thus, for a given deflection, Δ*d*, the ratio between the lateral sensitivity, $S_{l}=\;\frac{\Delta d}{\Delta V_{l}}$, and normal sensitivity, $S_{n}=\;\frac{\Delta d}{\Delta V_{n}}$, is

(8) $$\frac{S_{n}}{S_{l}}=\frac{\Delta V_{n}}{\Delta V_{l}}=\frac{\Delta \theta_{n}}{\Delta \theta_{l}}=\frac{\frac{3\cdot \Delta d}{2\cdot L}}{\frac{\Delta d}{\left(h+\frac{t}{2}\right)}}=\frac{3\cdot \left(h+\frac{t}{2}\right)}{2\cdot L}\;.$$

Therefore,

(9) $$S_{l}=\frac{3\cdot \left(h+\frac{t}{2}\right)}{2\cdot L}\cdot S_{n}$$

In the Asylum AFM MFP-3D-Bio, the normal sensitivity, *S_n_*, is termed InVols, and is recorded in m/V. In this AFM, the InVols (normal sensitivity) can be determined by two methods: calibration of the AFM tip, which provides an InVols value; and single touch. In the single-touch method, the tip is pressed on a hard surface (e.g., blank glass slide) and the sensitivity is recorded. The latter method is more direct and accurate, and was used here. However, the sensitivity values determined via the two methods were consistently within 10% of each other. 

Plugging Equations (2) and (9) into Equation (5), the equation for the lateral force is as follows:

(10) $$F_{l}=\frac{Gwt^{3}}{2L^{2}}\Delta V_{l}S_{n}$$

where $\Delta V_{l}$ is the measured lateral voltage signal, and *S_n_* is determined as described above. The shear modulus of silicon, G, has a value of 0.5 × 10^11^ Pa and the dimensions of the cantilever are provided by the cantilever manufacturer (and were verified using optical microscopy). However, the manufacturer-provided cantilever thickness, which enters the equation in the third power, has a large uncertainty. Thus, it was determined using Equation (1) for the normal spring constant, $k_{n}=\frac{E\cdot w\cdot t^{3}}{4\cdot L^{3}}$. The value for $k_{n}$ is determined from the equipartition theorem using built-in microscope software (calibration mentioned above): *L*, *w*, and *E* of the cantilever are known to high precision, so the thickness, *t*, can be extracted from this equation. This value is then used in Equation (10). The tip height, *h*, is also provided by the manufacturer and is 14 μm.

Each cantilever was calibrated before each experiment. We found the elastic, bent beam method described above is accurate with an overall error of 56% or less [41].

### 2.7. Fiber Stress–Strain Curves

The strain of the fiber, ε, is found by the change in the fiber length, Δ*L*, divided by the initial fiber length, *L_i_*, which can be represented by the equation

(11) $$\varepsilon =\;\frac{\left(L_{f}-L_{i}\right)}{L_{i}}\cdot 100\%$$

*L_f_*, the final length of the fiber, can be found using the Pythagorean Theorem (Figure 3b), $L_{f}=\sqrt{L_{i}{}^{2}+s^{2}}$. Since the gaps between ridges were of a fixed size, *L_i_* had a constant value of 3 μm (half of 6 μm, the gap between ridges). *L_f_* is the length of half of the extended fiber (assuming the fiber was pulled in the middle). 

The stress, *σ*, on a fiber with cross-sectional area, *A*, is defined as

(12) $$\sigma =\frac{F_{fiber}}{A}$$

where *F_fiber_* is the force exerted on the fiber along the length of the fiber by the tip. For these experiments, the diameter of the fiber was assumed to be constant (the initial value before stretching), as the fiber was stretched. In other words, we are ignoring the thinning of the fiber as it is stretched out (Poisson’s ratio μ = ∞). According to Newton’s third law, for every action force, there is an equal and opposite reaction force; therefore, *F_tip_* is equal and opposite to sum of the x-components of the force applied to the whole fiber, *−F_tip_*. Considering Figure 3c, and assuming the fiber is pulled in the middle, the force on one arm (one half) of the fiber is given by:

(13) $$F_{fiber}=\frac{F_{tip}}{2\cdot \sin\left(\beta \right)}=\frac{F_{l}}{2\cdot \sin\left(\beta \right)}$$

In the equation above, *β* is as the angle between the initial and final fiber position. Using Equation (10) above for the measured lateral force, *F_l_*, the force on the fiber can be calculated. Dividing it by the cross-sectional area of the fiber gives the stress on the fiber, Equation (12). 

Assuming a cylindrical fiber with diameter, *d*, the cross-section area, *A*, is $A=\frac{\pi }{4}d^{2}$ and combining Equations (12), (13) and (10), the stress on the fiber is

(14) $$\sigma =\;\frac{Gwt^{3}\Delta V_{l}S_{n}}{\sin\left(\beta \right)\pi d^{2}L^{2}}$$

All the quantities on the right side of the equation are known constants or can be measured. After determining the instantaneous stress and strain of the fibers, stress was plotted against strain and the slope of these curves was calculated to find the modulus of the single pulls. However, the slope was not constant, so the instantaneous slope was also calculated and plotted over the strain to show how the modulus of the fiber changes as a function of strain.

### 2.8. Incremental Stress–Strain Curves

For the incremental stress–strain curves, fibers were pulled incrementally (repeated pull, stop, wait cycles) and stress was plotted versus time. Individual relaxation curves of the fibers were then fitted to a double exponential function in Origin (OriginLab Corporation, Northampton, MA, USA) of stress versus times. The following equation was used:

(15) σ(t)= σ0+σ1∗e−tτ1+σ2∗e−tτ2

**Figure 4:**
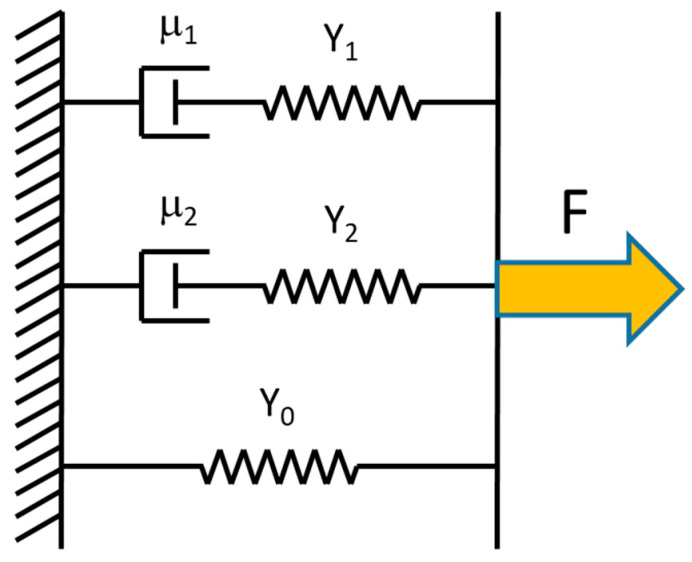
Kelvin model consisting of an elastic spring in parallel with two elements consisting of a dashpot and an elastic spring in series. This model was used to fit the incremental stress–strain curves.

This equation is based on a Kelvin model (Figure 4) of three mechanical elements in parallel: (1) an elastic spring with modulus *Y*_0_; (2) an elastic spring (modulus *Y*_1_) in series with a dashpot (viscosity *η*_1_); (3) an elastic spring (modulus *Y*_2_) in series with a dashpot (viscosity *η*_2_). All three elements are needed to fit the data. The first element is needed because the stress does not relax all the way to zero; there is a time-independent elastic component left as *t*→∞. The second and third elements are needed because the exponential decay shows two time regimes, a fast decay (*τ*_1_) and a slower decay (*τ*_2_). In Equation (15), *σ_0_* is the relaxed stress value of the fiber as *t*→∞. The coefficients σ_1_ and σ_2_ are values used to find the total modulus; *Y_t_ = Y_0_ + Y*_1_ *+ Y*_2_, where *Y_i_ = σ_i_/ε*, with *i* = 0, 1, 2. The values of $\tau_{1}$ and $\tau_{2}$ represent the fast and slow relaxation times, respectively. The relaxation time, viscosity and modulus of the in series elements are related by *τ_i_ = η_i_/Y_i_*, with *i* = 1, 2. 

Additionally, the energy loss, which is proportional to the area inscribed in a forward and return pull of a fiber, is calculated using midpoint Reimann sums. For the outward pull, the area under the curve can be calculated from the sum of the product of the average stress between two points, and the change in strain between the two data points,

(16) $$Area\;=\;\underset{i}{\sum }\left(\sigma_{i}+\sigma_{i+1}\right)\left(\frac{1}{2}\right)\left(\varepsilon_{i+1}-\varepsilon_{i}\right)$$

The unit of this quantity is energy per unit volume for stretching the fiber. The same calculation is done for the return trip, back to the starting point; here, some of the energy is recovered. 

The difference in area between the forward and return movement was calculated and divided by the area under the forward curve. This produced a percentage that represents the energy/volume lost in a cyclic stress–strain curve.

## 3. Results

**Figure 5:**
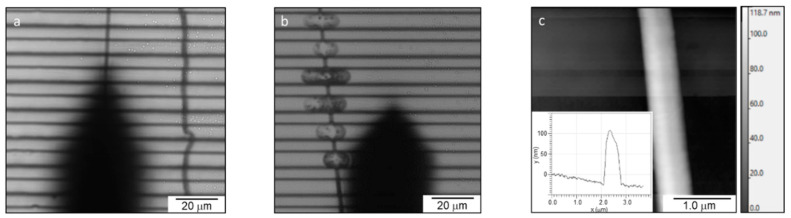
Electrospun 50:50 fibrinogen:PCL (poly-ε-caprolactone) fibers on striated substrate. (**a**) Optical microscopy image of two single fibers on the substrate with the AFM taking an image of one of the fibers. AFM probe is the black, pointed shape). (**b**) Optical microscopy image of an epoxy-anchored fiber. (**c**) AFM image of a single electrospun fiber on a ridge. The inset shows the cross-section of the fiber; fiber height (diameter), 135 nm.

The electrospun fibers were collected on a striated substrate on a glass slide. The fibers had an average diameter of 230 ± 90 nm. An image of the fibers roughly aligned perpendicularly on the ridges is shown in Figure 5a. The AFM was then used to scan the fibers on the ridges, providing images of individual fibers with an image size of 2 to 4 μm (Figure 5c). An anchored fiber is shown in Figure 5b. 

The AFM testing of fibers produces several different mechanical parameters that, together, provide a picture of the mechanical properties of the fiber.

### 3.1. Simple Stress–Strain Curves

**Figure 6:**
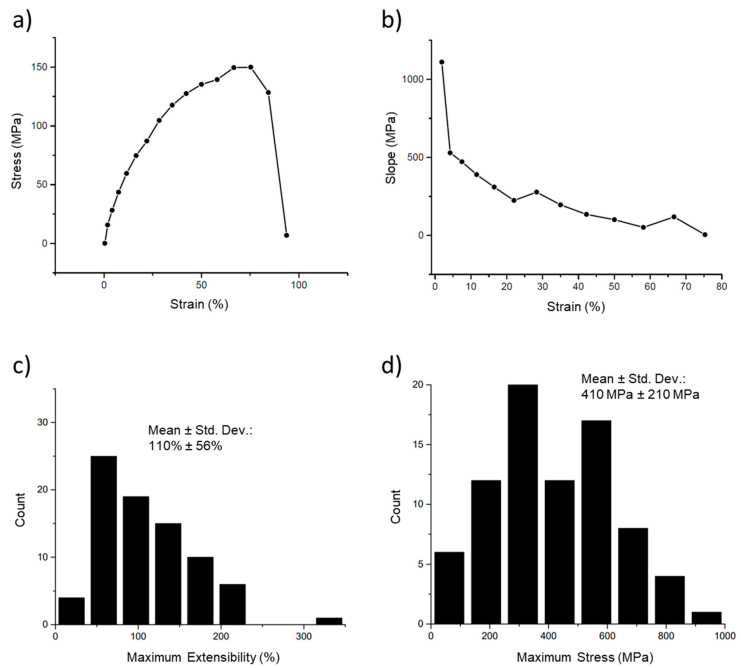
Stress–strain curve and modulus of an electrospun 50:50 fibrinogen:PCL fiber. (**a**) Stress–strain curve of a single fiber. Fiber stress increases nonlinearly with strain, initially with a steep slope that decreases continually, and plateaus between 67% and 75%, reaching a maximum stress of 150 MPa. The fiber ruptures between 84% and 94% strain. (**b**) A plot of the instantaneous slope (modulus) of the curve in (**a**) shows the continual decrease in modulus with increasing strain. (**c**) Histogram of maximum extensibility (maximum strain before rupture). The mean, around 110%, indicates the fiber is stretched to 2.1 times its initial length before breaking. (**d**) Histogram of rupture stress, the maximum stress before the fiber breaks.

The first tests conducted on the fibers are termed ‘simple’ stress–strain curves, in which a fiber is pulled until it ruptures and the stress is plotted versus the strain for this process (Figure 6a). This curve also shows the extreme strain softening of these fibers. Strain softening occurs when the fiber becomes softer (decreasing slope) the further it is stretched. Thus, at the higher strains, the stress increases more slowly, and the modulus (slope) is lower. This can be clearly seen in Figure 6b, in which the modulus (instantaneous slope) is plotted versus the strain. The strain softening was quite pronounced, with the modulus beginning at 1100 MPa but rapidly decreasing to a value of 100 MPa at 50% strain and approaching 0 Pa at strains close to the extensibility limit (breaking strain) of the fibers. Figure 6c,d show the distributions of the maximum extensibility (breaking strain) and maximum stress (highest stress before breaking).

### 3.2. Incremental Stress–Strain Curves

**Figure 7:**
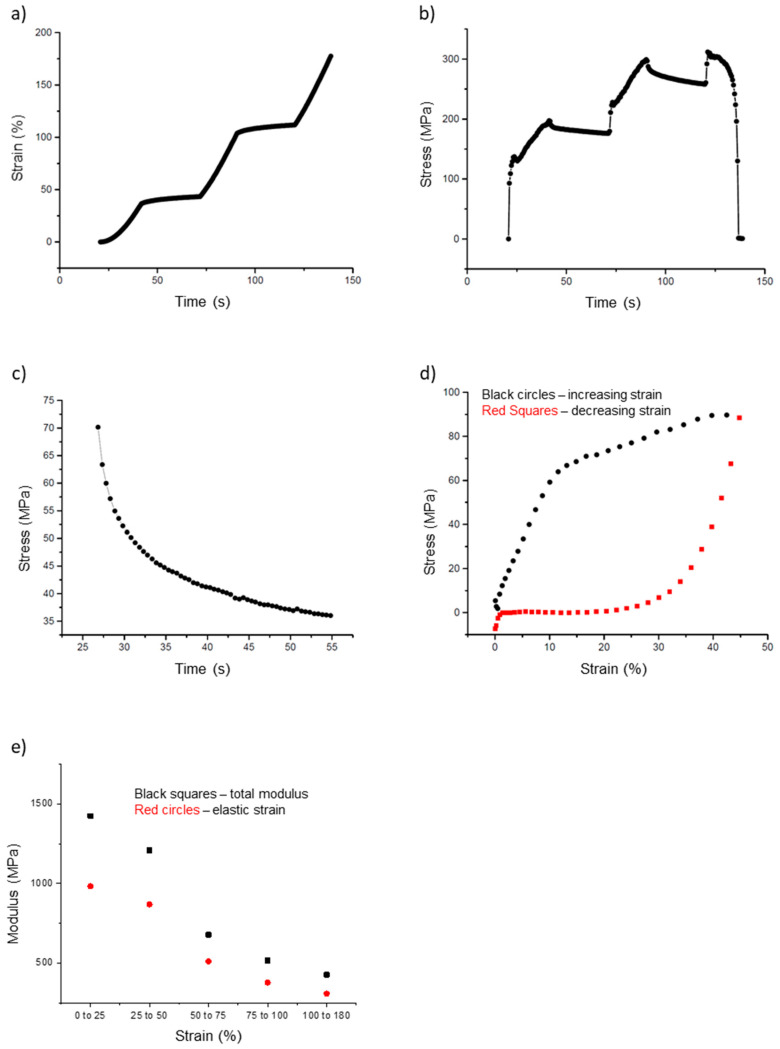
Incremental stress–strain curves and cyclical stress–strain curves of electrospun 50:50 fibrinogen:PCL fibers. (**a**) Strain versus time curve showing sequential periods of increasing strain (20 s) followed by constant strain (30 s). (**b**) Fiber stress versus time, fiber stress increases as the fiber is pulled; it decays during each constant strain period of the incremental stress-strain tests. (**c**) Close up of Figure 7b, showing a single stress relaxation curve of an incremental stress-strain test. Plotting stress versus time permits curve-fitting to Equation (15) to extract the total and elastic modulus and stress relaxation times, see main text. There is a steep initial decrease with a fast relaxation time, *τ*_1_, followed by a slower relaxation with relaxation time, *τ*_2_. (**d**) Cyclic stress–strain curve to determine energy loss and elastic limit. This graph represents one complete outward pull (black circles) and return (red squares); it highlights the large energy loss (inscribed area) experienced by the fibers. (**e**) Plot of the total and elastic moduli grouped by strain, as extracted from the incremental stress–strain curves. The data points represent averages for the strain intervals expressed on the x-axis.

In addition to the simple stress–strain curves, we also collected incremental stress–strain data (Figure 7a,b). In these tests, the fiber is pulled for a certain distance (typically 1000 nm; 20 s at 50 nm/s), and then allowed to relax for a certain amount of time (typically 30 s). This process is repeated until the fiber breaks. The purpose of these incremental curves is to separate the elastic (time independent) and the plastic (time-dependent) components of the fiber modulus. The stress on the fiber decreases during the 30 s of relaxation, which indicates a time-dependent, plastic component of the fiber modulus. 

In Figure 7a, the strain increases from 20 to 40 s, from 70 to 90 s, and 120 to 140 s, at which point the fiber breaks. In between these pulls, the strain on the fiber is held constant (tip movement is halted). However, as seen in Figure 7b, the stress decreases during these same periods of constant strain. These portions, called stress relaxation curves, were fit to a double exponential (Equation (15), Figure 7c). These fits were then used to calculate the total and elastic moduli and the stress relaxation times.

For the incremental stress–strain test, the moduli calculated at each successive incremental curve decreased as the strain increased. The total modulus values had values around 1400 MPa at strain values < 25%, but decreased to 430 MPa at a strain around 100%. Likewise, the elastic modulus had values around 980 MPa at a strain of 25% and decreased to around 310 MPa at 100% strain. The ratio of elastic to total modulus remained around 0.7 as strain increased from small to large values, indicating elastic and plastic behavior of the fiber. This can also be seen in Figure 7e, in which the total and elastic moduli are grouped by strain and plotted. Both moduli values decrease as strain increases. The key mechanical parameters extracted from the incremental stress–strain curves are summarized in Table 1. 

### 3.3. Energy Loss and Elastic Limit

The final tests were cyclical stress–strain curves to determine the elastic limit and energy loss; the test involves pulling the fiber to a certain strain and then returning the tip to the starting position (Figure 7d). This process was repeated several times for each fiber with increasing strain values, until the fiber broke. These data can be used to determine the energy lost due to plastic deformations. The energy loss is the inscribed area in a cyclical stress–strain curve. The outward curve, depicted in black in Figure 7d, follows the shape of a typical stress–strain curve (Figure 6a). However, as the tip returns to its initial position (red curve), the stretched fiber does not return to its initial position. This can be inferred from the stress values of the fiber. In Figure 7d, the red curve, moving from right to left, returns to a value of 0 MPa around 20%. This means the fiber is slack at this point and no longer pulling on the tip. 

## 4. Discussion

We determined the mechanical properties of single, electrospun fibers made from a 50:50 blend of fibrinogen and PCL. A summary of the key mechanical parameters obtained from the simple, incremental, and cyclical stress–strain curves is given in Table 1. 

The large standard deviation for many of these categories follows patterns found in previous research in this area [31,32,33,34]. Although the relaxation curves that produced the data fit well to Equation (15), with a typical value of 0.98 for R^2^, the values obtained for total and elastic moduli have large standard deviations, which is indicative of the large variability across fibers. 

In both the simple stress–strain curves and the incremental stress–strain curves significant strain softening (a strong decrease in modulus as strain is increased) is observed. In the simple stress–strain curves the modulus decreases from 1700 MPa at 10% strain to 110 MPa at 100% strain; similarly in the incremental stress–strain curves the total modulus decreases from 1400 MPa at 10% strain to 310 MPa at 100% strain. Cyclical stress–strain curves indicate that the elastic limit is very low, 5%, and the energy loss is very high, indicative of largely plastic deformations. 

It is instructive to compare the mechanical properties of the blended fibrinogen: PCL fibers with those of the electrospun, pure PCL and pure fibrinogen fibers [31,32,34], and also other electrospun and natural fibers (Table 2). Dry electrospun fibrinogen fibers have a total and elastic modulus of 4200 MPa and 3700 MPa, respectively. They have an extensibility of 110% and an elastic limit of 16%, they also showed significant strain softening, as the modulus decreased by a factor of 3 at about 20% strain [32]. This decrease is sudden and not gradual as seen in the blended fibers [32]. The energy loss was not recorded in this work; however it can be estimated to be about 60% at 13% strain from Figure 4 in reference [32]. At larger strains it is likely larger, around 70. Electrospun PCL fibers have a total and elastic modulus of 62 MPa and 53 MPa, respectively [31]. Other groups have reported somewhat higher values for the modulus of PCL fibers, which may be due to different fiber preparations [43,44,45,46,47]. Electrospun PCL fibers have an extensibility of 98% and an elastic limit of 19%. They also showed significant strain softening, as the modulus decreases by a factor of 7 at about 30% strain. In cyclic stress–strain curves they show increasing energy loss with increasing strain, from about 20% energy at 10% strain to nearly 60% energy loss at larger than 45% strain [31].

The fibrinogen and PCL solutions in HFP were well miscible, without any apparent separation or precipitation. However, it was not clear how the mechanical properties of the blended fibers would relate to those of the ‘pure’ fibers, especially given that fibrinogen and PCL have completely different molecular structures. We found that despite these structural differences, the blended fibers showed high structural integrity with mechanical properties that were between the properties of the ‘pure’ fibers, though somewhat closer to the fibrinogen fibers. The value of the total modulus of the blended fibers (1100 MPa from incremental stress strain curves) is only 3.8 times smaller but 18 times larger than the modulus of the ‘pure’ fibrinogen and PCL fibers, respectively. This may indicate that the modulus is dominated by the fibrinogen component of the blended fibers, and that intermolecular connections between the fibrinogen molecules still exist but may be weakened or lost due to the presence of PCL. The extensibility of the blended fiber (110%) is very similar to that of the pure fibers (fibrinogen, 113%; PCL, 98%). The elastic limit of the blended fibers (5%) is somewhat lower than the values of the ‘pure’ fibers (fibrinogen 16%; PCL, 19%). This is an indication that there are fewer elastic bonds between the components of the blended fibers, as compared to the ‘pure’ fibers. The energy loss for the blended and for the ‘pure’ fibers is very large, around 60% to 70%, indicative of significant plastic deformations; these electrospun fibers all display the stress–strain curves of a ductile material. Using an AFM-based nanoindentation method a similarly large energy loss was found for a single electrospun polyvinylidene fluoride (PVDF) nanofibers; the modulus for these fibers was 2200 MPa [48].

Table 2 also lists the mechanical properties of fibrin fibers, collagen fibers and electrospun collagen fibers. Fibrin fibers are particularly relevant because they are the natural fibers formed by fibrinogen. Upon activation by thrombin, fibrinogen forms a network of 100–200 nm thick fibrin fibers, which is the major structural and mechanical component of blood clots. Fibrinogen has a nearly 400 amino acid long, largely unstructured region (the alphaC region), which internally connects molecules and protofibrils together [49]. It could well be that these unfolded regions may also form connections in electrospun fibrinogen fibers and in fibrinogen: PCL fibers. In the fibrinogen: PCL fibers these unfolded domains may also get entangled with the long PCL polymer chain. In contrast, these unfolded domains are missing in collagen fibers, which have a much lower extensibility (native and electrospun collagen).

Very recently, researchers formed vascular grafts from electrospun, blended fibrinogen:PCL fibers. The grafts showed robust mechanical properties and biomimetic arrangement of extracellular matrix (ECM) with rich expression of elastic fiber-related proteins [53]. These results indicate the promise of using blended, electrospun fibers.

Our data on the mechanical properties of electrospun 50:50 fibrinogen:PCL fibers expand the library of mechanically characterized fibers available for biomedical applications. The 50:50 fibers show the properties of a ductile material. Future studies may focus on uncovering the molecular mechanism that give rise to these mechanical properties.

## Figures and Tables

**Table 1:** Summary of the data collected in this paper. We followed the recommendation to use one decimal place more than the precision of our measurement [42]. We estimate the absolute error in our force measurement to be about 50% and in our extensibility measurement to be about 10%, given the error in the measured, experimental quantities. The high standard deviation of these values reflects the large variability across different fibers, not necessarily the error in measurements.

Value	Average +/− Std. Dev.	Data Points, N
Extensibility (Maximum Strain)	110 ± 60%	80
Maximum Stress	410 ± 210 MPa	86
Elastic Limit	5 ± 5%	51
Energy Loss	75 ± 10%	25
Fast Relaxation Time	1.1 ± 0.4 s	87
Slow Relaxation Time	16 ± 6 s	86
Initial Modulus, single pull (~10% strain)	1700 ± 800 MPa	9
Large strain Modulus, single pull (~100% strain)	110 ± 90 MPa	9
Total Modulus, incremental (0–25% strain)	1400 ± 990 MPa	90
Elastic Modulus, incremental (0–25% strain)	980 ± 710 MPa	90
Total Modulus, incremental (~100% strain)	430 ± 420 MPa	90
Elastic Modulus, incremental (~100% strain)	310 ± 250 MPa	90

**Table 2:** Comparison of select properties from some relevant electrospun and natural fibers.

Fiber Type	Extensibility (%)	Fast Relax (s)	Slow Relax (s)	Elastic Modulus (MPa)	Total Modulus (MPa)	Diameter (nm)
e-spun 50:50 Fibrinogen:PCL [this paper]	110 ± 60	1.1 ± 0.4	16 ± 6	980 ± 710 (low strain values)	1400 ± 990 (low strain values)	230 ± 90
e-spun PCL Fibers (>30 days) [31]	98 ± 30	0.98 ± 0.26	8.79 ± 3.08	52.9 ± 36.2	62.3 ± 25.6	440–1040
e-spun PCL Fibers (<30 days) [31]		1.69 ± 0.44	21.22 ± 8.97	61.4 ± 51.1	99.2 ± 83.9	440–1040
e-spun Fibrinogen (dry) [32]	113 ± 22	1.2 ± 0.4	11 ± 5	4200 ± 3400	3700 ± 3100	30–200
Native Collagen [50]	12				160–7500	(tendon)
Cross-linked Fibrin [51]	147	2.1	49	8.0	4.0	124–800
Uncrosslinked Fibrin [51]	226	2.9	54	3.9	1.9	94–700
Dry e-spun Collagen Fibers [33]	33 ± 3	3	50		2800 ± 3100	200–800
Spider Silk [52]	270				3	1000–5000
